# Crystal structure of 3-(hy­droxy­meth­yl)chromone

**DOI:** 10.1107/S2056989015011627

**Published:** 2015-06-20

**Authors:** Yoshinobu Ishikawa

**Affiliations:** a School of Pharmaceutical Sciences, University of Shizuoka, 52-1 Yada, Suruga-ku, Shizuoka 422-8526, Japan

**Keywords:** crystal structure, chromone, hydrogen bonding, π–π stacking

## Abstract

In the title compound, C_10_H_8_O_3_ (systematic name 3-hy­droxy­methyl-4*H*-chromen-4-one), the fused-ring system is slightly puckered [dihedral angle between the rings = 3.84 (11)°]. The hy­droxy O atom deviates from the heterocyclic ring by 1.422 (1) Å. In the crystal, inversion dimers linked by pairs of O—H⋯O hydrogen bonds generate *R*_2_^2^(12) loops. The dimers are linked by aromatic π–π stacking [shortest centroid–centroid distance = 3.580 (3) Å], and C—H⋯O hydrogen bonds, generating a three-dimensional network.

## Related literature   

For the biological activities of related compounds, see: Sun *et al.* (2009 ▸); Helguera *et al.* (2013 ▸); Venkateswararao *et al.* (2014 ▸). For the synthesis of the title compound, see: Araya-Maturana *et al.* (2003 ▸).
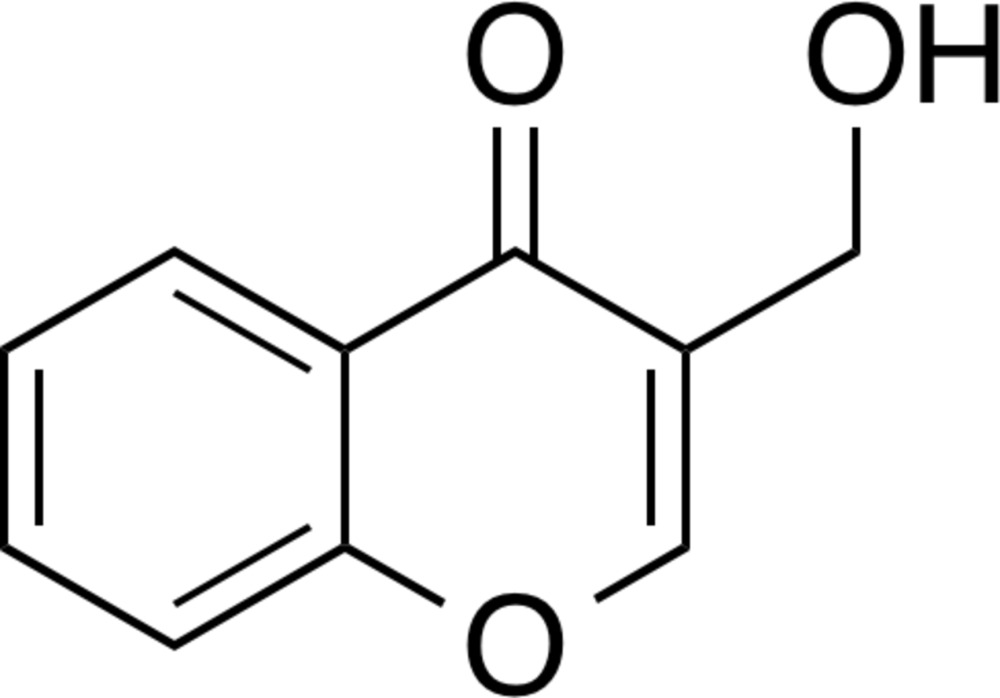


## Experimental   

### Crystal data  

C_10_H_8_O_3_
*M* *_r_* = 176.17
Triclinic, 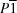

*a* = 6.756 (4) Å
*b* = 7.988 (6) Å
*c* = 7.991 (6) Å
α = 94.48 (6)°
β = 108.27 (5)°
γ = 103.31 (5)°
*V* = 393.2 (5) Å^3^
*Z* = 2
Mo *K*α radiation
μ = 0.11 mm^−1^
*T* = 100 K
0.32 × 0.32 × 0.16 mm

### Data collection  

Rigaku AFC-7R diffractometer
2219 measured reflections
1805 independent reflections
1537 reflections with *F* ^2^ > 2.0σ(*F* ^2^)
*R*_int_ = 0.089
3 standard reflections every 150 reflections intensity decay: 0.1%

### Refinement  

*R*[*F* ^2^ > 2σ(*F* ^2^)] = 0.065
*wR*(*F* ^2^) = 0.202
*S* = 1.06
1805 reflections
119 parameters
H-atom parameters constrained
Δρ_max_ = 0.42 e Å^−3^
Δρ_min_ = −0.49 e Å^−3^

### 

Data collection: *WinAFC Diffractometer Control Software* (Rigaku, 1999 ▸); cell refinement: *WinAFC Diffractometer Control Software*; data reduction: *WinAFC Diffractometer Control Software*; program(s) used to solve structure: *SIR2008* (Burla *et al.*, 2007 ▸); program(s) used to refine structure: *SHELXL97* (Sheldrick, 2008 ▸); molecular graphics: *CrystalStructure* (Rigaku, 2010 ▸); software used to prepare material for publication: *CrystalStructure*.

## Supplementary Material

Crystal structure: contains datablock(s) General, I. DOI: 10.1107/S2056989015011627/hb7444sup1.cif

Structure factors: contains datablock(s) I. DOI: 10.1107/S2056989015011627/hb7444Isup2.hkl

Supporting information file. DOI: 10.1107/S2056989015011627/hb7444Isup3.cml

**Fig. 1.**
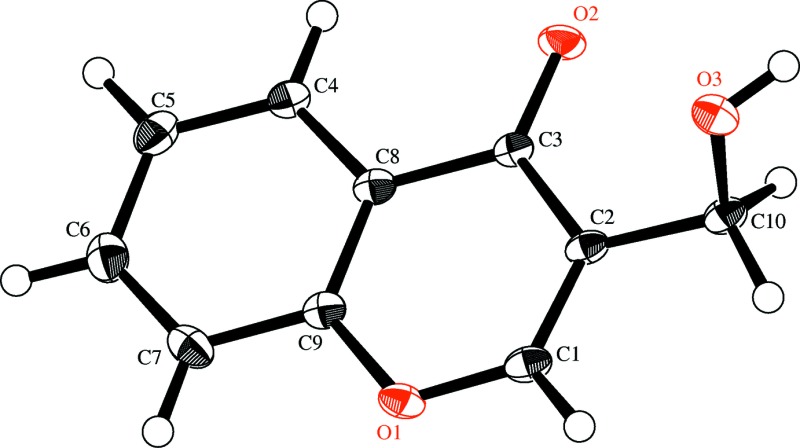
The molecular structure of the title compound, with displacement ellipsoids drawn at the 50% probability level.

. DOI: 10.1107/S2056989015011627/hb7444fig1.tif
The mol­ecular structure of the title compound, with displacement ellipsoids drawn at the 50% probability level.

**Fig. 2.**
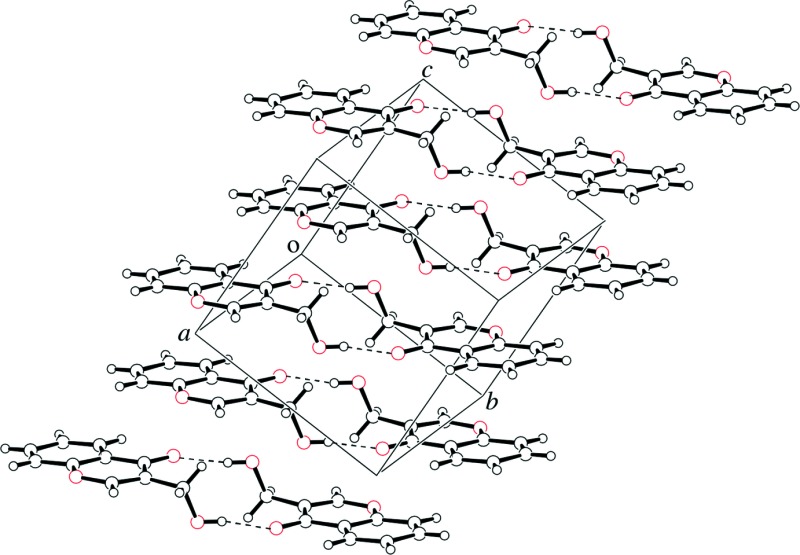
A view of the packing of the title compound. O—H···O hydrogen bonds are represented as dashed lines.

. DOI: 10.1107/S2056989015011627/hb7444fig2.tif
A view of the packing of the title compound. O—H⋯O hydrogen bonds are represented as dashed lines.

CCDC reference: 1406927

Additional supporting information:  crystallographic information; 3D view; checkCIF report

## Figures and Tables

**Table 1:** Hydrogen-bond geometry (, )

*D*H*A*	*D*H	H*A*	*D* *A*	*D*H*A*
O3H8O2^i^	0.84	1.94	2.757(3)	165
C1H1O2^ii^	0.95	2.58	3.283(4)	131

Symmetry codes: (i) 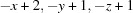; (ii) 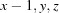.

## supplementary crystallographic information

### S1. Comment

Many derivatives of the title compound (3-hydroxymethylchromone) are reported as retinoic acid receptor binders (Sun *et al.* (2009)), human monoamine oxidase inhibitors (Helguera *et al.* (2013)) and anti-proliferative agents (Venkateswararao *et al.* (2014)).

The mean deviation of the least-square planes for the non-hydrogen atoms except hydroxy O3 atom is 0.0479 Å, and the largest deviation is 0.146 (2) Å for C10. These mean that these atoms are essentially coplanar (Fig.1). The dihedral angle of C3–C2–C10–O3 is 70.6 (2). In the crystal, the pyran rings are stacked [centroid–centroid distance between the pyran rings of the 4*H*-chromene units = 3.894 (3) Å], and C–H···O hydrogen bonds are formed to give dimers running along the *c* direction, as shown in Fig.2.

### S2. Experimental

The title compound was synthesized from 3-formylchromone according to the literature method (Araya-Maturana *et al.* 2003). Colourless blocks were obtained by slow evaporation of an ethyl acetate solution of the title compound at room temperature.

### S3. Refinement

All hydrogen atoms were placed in geometrical positions [C–H 0.95 Å and O–H 0.84 Å], and refined using a riding model with *U*_iso_(H) = 1.2*U*_eq_ of the parent atoms.

### Crystal data

**Table:**

C_10_H_8_O_3_	*Z* = 2
*M**_r_* = 176.17	*F*(000) = 184.00
Triclinic, *P*1	*D*_x_ = 1.488 Mg m^−^^3^
Hall symbol: -P 1	Mo *K*α radiation, λ = 0.71069 Å
*a* = 6.756 (4) Å	Cell parameters from 25 reflections
*b* = 7.988 (6) Å	θ = 15.5–17.3°
*c* = 7.991 (6) Å	µ = 0.11 mm^−^^1^
α = 94.48 (6)°	*T* = 100 K
β = 108.27 (5)°	Block, colorless
γ = 103.31 (5)°	0.32 × 0.32 × 0.16 mm
*V* = 393.2 (5) Å^3^	

### Data collection

**Table:**

Rigaku AFC-7R diffractometer	θ_max_ = 27.5°
ω–2θ scans	*h* = −4→8
2219 measured reflections	*k* = −10→10
1805 independent reflections	*l* = −10→9
1537 reflections with *F*^2^ > 2.0σ(*F*^2^)	3 standard reflections every 150 reflections
*R*_int_ = 0.089	intensity decay: 0.1%

### Refinement

**Table:**

Refinement on *F*^2^	Secondary atom site location: difference Fourier map
*R*[*F*^2^ > 2σ(*F*^2^)] = 0.065	Hydrogen site location: inferred from neighbouring sites
*wR*(*F*^2^) = 0.202	H-atom parameters constrained
*S* = 1.06	*w* = 1/[σ^2^(*F*_o_^2^) + (0.1399*P*)^2^ + 0.1723*P*] where *P* = (*F*_o_^2^ + 2*F*_c_^2^)/3
1805 reflections	(Δ/σ)_max_ < 0.001
119 parameters	Δρ_max_ = 0.42 e Å^−^^3^
0 restraints	Δρ_min_ = −0.49 e Å^−^^3^
Primary atom site location: structure-invariant direct methods	

### Special details

**Table:**

Refinement. Refinement was performed using all reflections. The weighted *R*-factor (*wR*) and goodness of fit (*S*) are based on *F*^2^. *R*-factor (gt) are based on *F*. The threshold expression of *F*^2^ > 2.0 *σ*(*F*^2^) is used only for calculating *R*-factor (gt).

### Fractional atomic coordinates and isotropic or equivalent isotropic displacement parameters (Å^2^)

**Table:**

	*x*	*y*	*z*	*U*_iso_*/*U*_eq_	
O1	0.72779 (19)	1.01991 (17)	0.70911 (18)	0.0179 (4)	
O2	1.19522 (19)	0.80874 (17)	0.63213 (18)	0.0190 (4)	
O3	0.7925 (2)	0.48525 (17)	0.56522 (17)	0.0195 (4)	
C1	0.6785 (3)	0.8666 (3)	0.6006 (3)	0.0167 (4)	
C2	0.8224 (3)	0.7871 (3)	0.5707 (3)	0.0146 (4)	
C3	1.0527 (3)	0.8696 (3)	0.6575 (3)	0.0133 (4)	
C4	1.3221 (3)	1.1214 (3)	0.8824 (3)	0.0168 (4)	
C5	1.3681 (3)	1.2688 (3)	1.0053 (3)	0.0209 (5)	
C6	1.1985 (3)	1.3325 (3)	1.0269 (3)	0.0218 (5)	
C7	0.9868 (3)	1.2504 (3)	0.9255 (3)	0.0200 (5)	
C8	1.1070 (3)	1.0325 (3)	0.7810 (3)	0.0147 (4)	
C9	0.9416 (3)	1.0995 (3)	0.8033 (3)	0.0153 (4)	
C10	0.7477 (3)	0.6132 (3)	0.4539 (3)	0.0165 (4)	
H1	0.5294	0.8101	0.5403	0.0201*	
H2	1.4369	1.0798	0.8665	0.0202*	
H3	1.5139	1.3272	1.0751	0.0251*	
H4	1.2306	1.4333	1.1123	0.0262*	
H5	0.8729	1.2955	0.9382	0.0240*	
H6A	0.5905	0.5855	0.3870	0.0198*	
H7B	0.8247	0.6145	0.3669	0.0198*	
H8	0.8200	0.4052	0.5093	0.0234*	

### Atomic displacement parameters (Å^2^)

**Table:**

	*U*^11^	*U*^22^	*U*^33^	*U*^12^	*U*^13^	*U*^23^
O1	0.0130 (6)	0.0202 (7)	0.0240 (7)	0.0070 (5)	0.0090 (5)	0.0043 (5)
O2	0.0112 (6)	0.0225 (7)	0.0252 (7)	0.0067 (5)	0.0075 (5)	0.0032 (6)
O3	0.0195 (7)	0.0188 (7)	0.0236 (7)	0.0069 (5)	0.0104 (6)	0.0052 (5)
C1	0.0110 (8)	0.0212 (9)	0.0193 (9)	0.0041 (7)	0.0061 (7)	0.0073 (7)
C2	0.0108 (8)	0.0202 (9)	0.0144 (8)	0.0046 (7)	0.0050 (6)	0.0067 (7)
C3	0.0115 (8)	0.0168 (9)	0.0129 (8)	0.0043 (6)	0.0052 (6)	0.0053 (6)
C4	0.0158 (8)	0.0179 (9)	0.0168 (8)	0.0054 (7)	0.0044 (7)	0.0050 (7)
C5	0.0205 (9)	0.0212 (10)	0.0174 (9)	0.0040 (7)	0.0021 (7)	0.0055 (7)
C6	0.0302 (10)	0.0184 (9)	0.0171 (9)	0.0071 (8)	0.0077 (8)	0.0042 (7)
C7	0.0264 (9)	0.0198 (9)	0.0212 (9)	0.0113 (8)	0.0136 (8)	0.0068 (7)
C8	0.0144 (8)	0.0179 (9)	0.0140 (8)	0.0055 (7)	0.0061 (7)	0.0071 (7)
C9	0.0149 (8)	0.0191 (9)	0.0144 (8)	0.0055 (7)	0.0068 (7)	0.0069 (7)
C10	0.0101 (8)	0.0204 (9)	0.0178 (9)	0.0026 (7)	0.0039 (6)	0.0038 (7)

### Geometric parameters (Å, º)

**Table:**

O1—C1	1.352 (3)	C6—C7	1.375 (3)
O1—C9	1.371 (2)	C7—C9	1.400 (3)
O2—C3	1.236 (3)	C8—C9	1.396 (3)
O3—C10	1.429 (3)	O3—H8	0.840
C1—C2	1.346 (3)	C1—H1	0.950
C2—C3	1.455 (3)	C4—H2	0.950
C2—C10	1.495 (3)	C5—H3	0.950
C3—C8	1.468 (3)	C6—H4	0.950
C4—C5	1.381 (3)	C7—H5	0.950
C4—C8	1.404 (3)	C10—H6A	0.990
C5—C6	1.407 (4)	C10—H7B	0.990
			
C1—O1—C9	117.98 (17)	O3—C10—C2	108.17 (15)
O1—C1—C2	125.63 (15)	C10—O3—H8	109.472
C1—C2—C3	119.38 (17)	O1—C1—H1	117.186
C1—C2—C10	120.66 (15)	C2—C1—H1	117.188
C3—C2—C10	119.93 (18)	C5—C4—H2	119.761
O2—C3—C2	123.48 (17)	C8—C4—H2	119.765
O2—C3—C8	121.37 (15)	C4—C5—H3	120.061
C2—C3—C8	115.15 (18)	C6—C5—H3	120.064
C5—C4—C8	120.5 (2)	C5—C6—H4	119.658
C4—C5—C6	119.88 (16)	C7—C6—H4	119.653
C5—C6—C7	120.69 (19)	C6—C7—H5	120.506
C6—C7—C9	119.0 (3)	C9—C7—H5	120.503
C3—C8—C4	121.64 (19)	O3—C10—H6A	110.064
C3—C8—C9	119.77 (15)	O3—C10—H7B	110.063
C4—C8—C9	118.56 (17)	C2—C10—H6A	110.069
O1—C9—C7	116.70 (19)	C2—C10—H7B	110.064
O1—C9—C8	121.91 (17)	H6A—C10—H7B	108.407
C7—C9—C8	121.38 (16)		
			
C1—O1—C9—C7	−174.88 (15)	C5—C4—C8—C3	−176.20 (17)
C1—O1—C9—C8	4.2 (3)	C5—C4—C8—C9	1.9 (3)
C9—O1—C1—C2	−2.8 (3)	C8—C4—C5—C6	−1.2 (3)
C9—O1—C1—H1	177.2	C8—C4—C5—H3	178.8
H8—O3—C10—C2	−148.1	H2—C4—C5—C6	178.8
H8—O3—C10—H6A	91.6	H2—C4—C5—H3	−1.2
H8—O3—C10—H7B	−27.8	H2—C4—C8—C3	3.8
O1—C1—C2—C3	−1.0 (3)	H2—C4—C8—C9	−178.1
O1—C1—C2—C10	177.18 (16)	C4—C5—C6—C7	−0.7 (3)
H1—C1—C2—C3	179.0	C4—C5—C6—H4	179.3
H1—C1—C2—C10	−2.8	H3—C5—C6—C7	179.3
C1—C2—C3—O2	−177.19 (17)	H3—C5—C6—H4	−0.7
C1—C2—C3—C8	3.3 (3)	C5—C6—C7—C9	1.6 (3)
C1—C2—C10—O3	−107.56 (19)	C5—C6—C7—H5	−178.4
C1—C2—C10—H6A	12.7	H4—C6—C7—C9	−178.4
C1—C2—C10—H7B	132.2	H4—C6—C7—H5	1.6
C3—C2—C10—O3	70.6 (2)	C6—C7—C9—O1	178.25 (17)
C3—C2—C10—H6A	−169.1	C6—C7—C9—C8	−0.8 (3)
C3—C2—C10—H7B	−49.7	H5—C7—C9—O1	−1.8
C10—C2—C3—O2	4.6 (3)	H5—C7—C9—C8	179.2
C10—C2—C3—C8	−174.93 (15)	C3—C8—C9—O1	−1.8 (3)
O2—C3—C8—C4	−3.4 (3)	C3—C8—C9—C7	177.23 (16)
O2—C3—C8—C9	178.52 (16)	C4—C8—C9—O1	−179.96 (16)
C2—C3—C8—C4	176.19 (15)	C4—C8—C9—C7	−0.9 (3)
C2—C3—C8—C9	−1.9 (3)		

### Hydrogen-bond geometry (Å, º)

**Table:**

*D*—H···*A*	*D*—H	H···*A*	*D*···*A*	*D*—H···*A*
O3—H8···O2^i^	0.84	1.94	2.757 (3)	165
C1—H1···O2^ii^	0.95	2.58	3.283 (4)	131

Symmetry codes: (i) −*x*+2, −*y*+1, −*z*+1; (ii) *x*−1, *y*, *z*.
