# Supplementary figures and images for: Crystal structure of 3-(hy­droxy­meth­yl)chromone
Source: Acta Crystallogr E Crystallogr Commun. 2015 Jun 20;71(Pt 7):o495. doi: 10.1107/S2056989015011627 (PMC4518918; doi:10.1107/S2056989015011627)

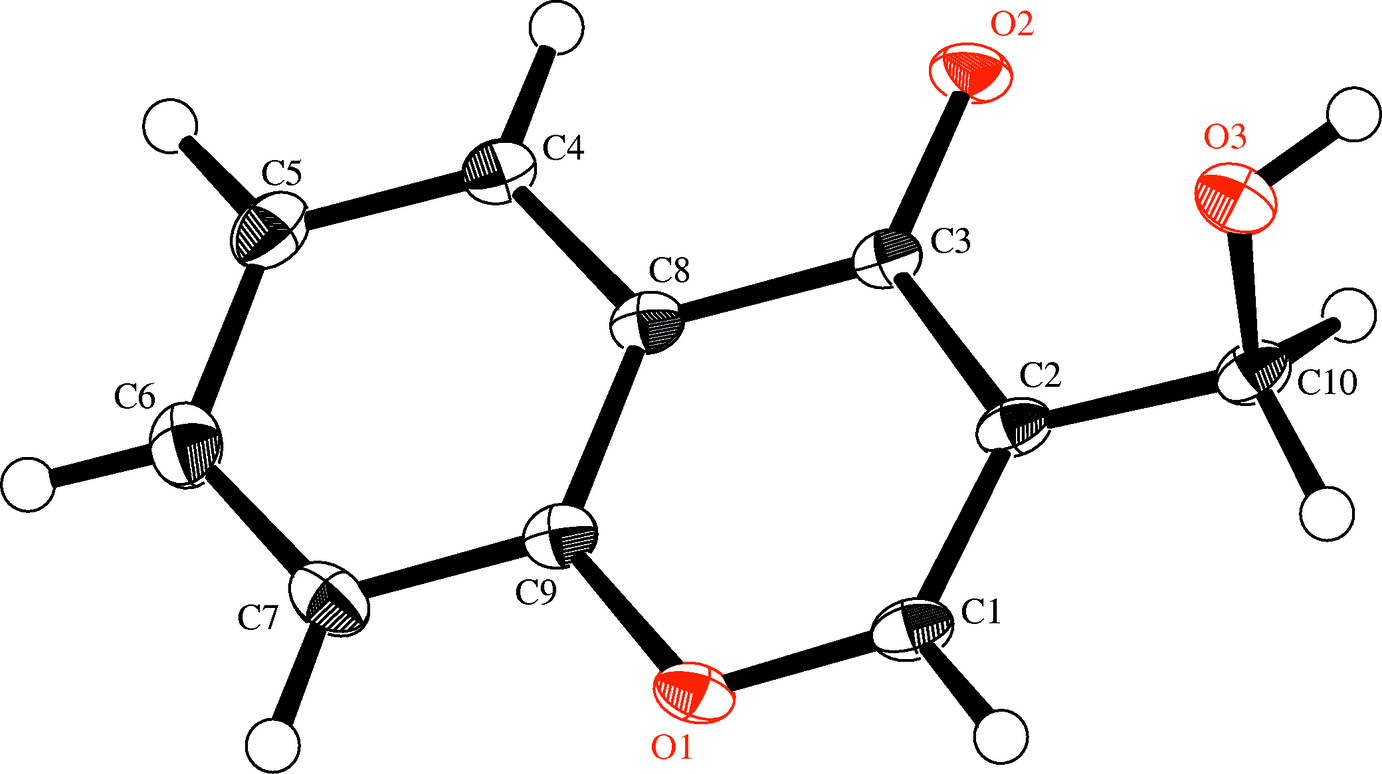

Supplement: Supplementary file 4 [file e-71-0o495-fig1.tif]

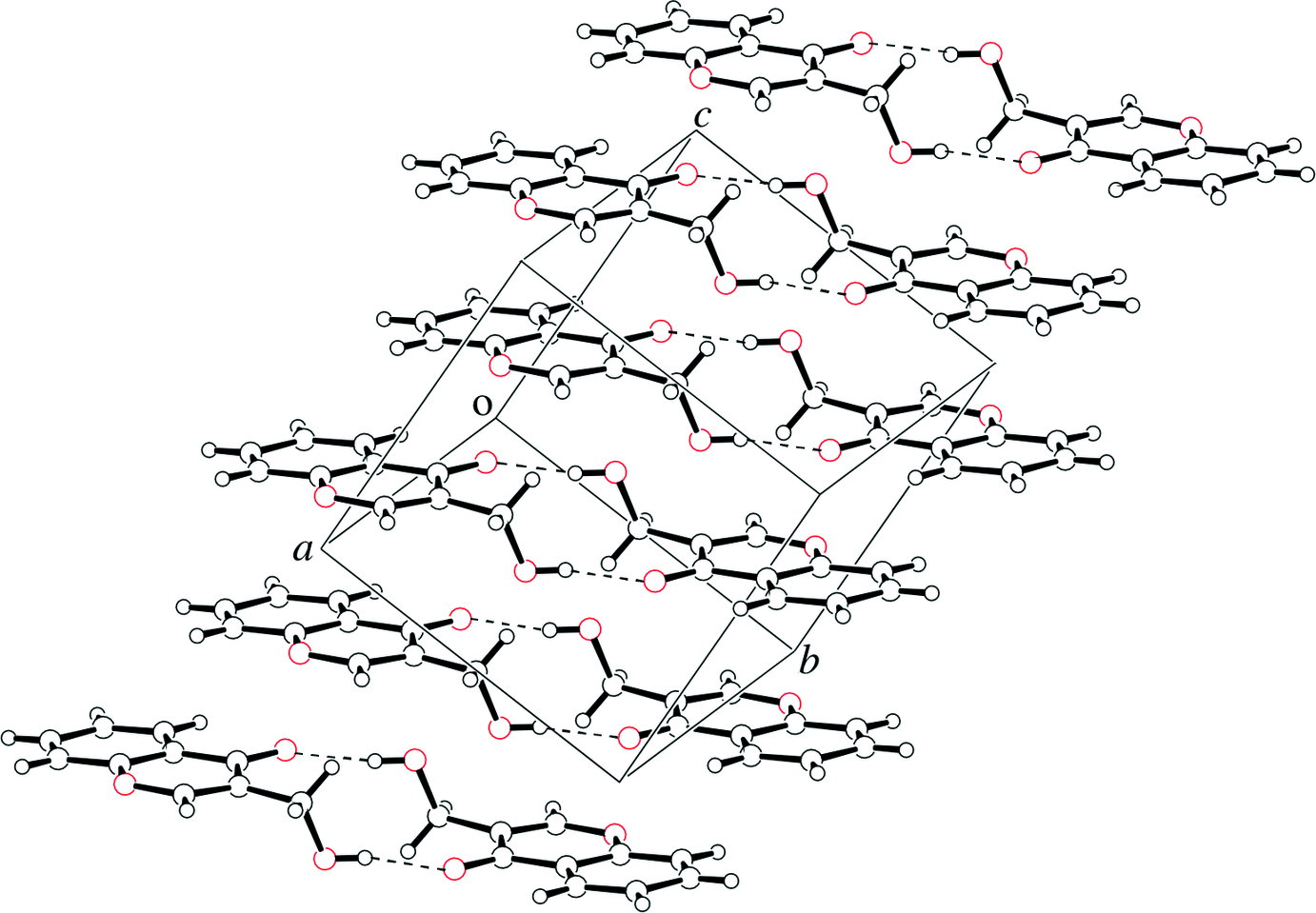

Supplement: Supplementary file 5 [file e-71-0o495-fig2.tif]
